# Targeting dysregulated intracellular immunometabolism within synovial microenvironment in rheumatoid arthritis with natural products

**DOI:** 10.3389/fphar.2024.1403823

**Published:** 2024-07-22

**Authors:** Shengtao Hu, Ye Lin, Yuanyuan Tang, Junlan Zhang, Yini He, Gejing Li, Liqing Li, Xiong Cai

**Affiliations:** ^1^ Institute of Innovation and Applied Research in Chinese Medicine, Hunan University of Chinese Medicine, Changsha, Hunan, China; ^2^ The Central Research Laboratory, Hunan Traditional Chinese Medical College, Zhuzhou, Hunan, China

**Keywords:** natural products, rheumatoid arthritis, glycolysis, lipid metabolism, amino acid metabolism, nucleotide metabolism, immunometabolic balance

## Abstract

Immunometabolism has been an emerging hotspot in the fields of tumors, obesity, and atherosclerosis in recent decades, yet few studies have investigated its connection with rheumatoid arthritis (RA). In principle, intracellular metabolic pathways upstream regulated by nutrients and growth factors control the effector functions of immune cells. Dynamic communication and hypermetabolic lesions of immune cells within the inflammatory synovial microenvironment contributes to the development and progression of RA. Hence, targeting metabolic pathways within immune subpopulations and pathological cells may represent novel therapeutic strategies for RA. Natural products constitute a great potential treasury for the research and development of novel drugs targeting RA. Here, we aimed to delineate an atlas of glycolysis, lipid metabolism, amino acid biosynthesis, and nucleotide metabolism in the synovial microenvironment of RA that affect the pathological processes of synovial cells. Meanwhile, therapeutic potentials and pharmacological mechanisms of natural products that are demonstrated to inhibit related key enzymes in the metabolic pathways or reverse the metabolic microenvironment and communication signals were discussed and highlighted.

## Graphical Abstract

**Figure:**
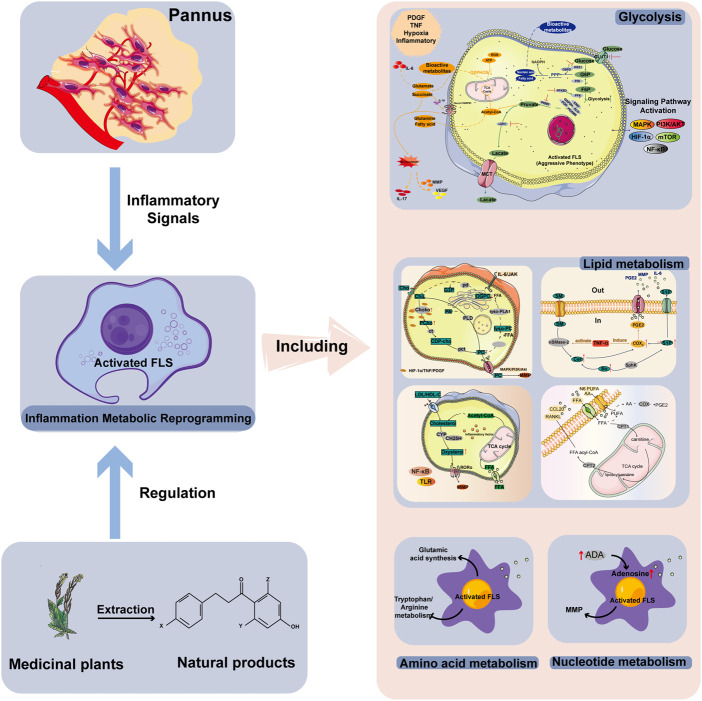
Activated FLSs in the synovial microenvironment of RA will undergo metabolic reprogramming of glycolysis, lipid, amino acid and nucleotide metabolism under the stimulation of inflammation, hypoxia and other conditions. The bioactive natural products of medicinal plants are able to regulate these four major metabolic modes to reverse the abnormal metabolism in the RA microenvironment.

## 1 Introduction

Rheumatoid arthritis (RA) is a disease caused by the continuous proliferation of fibroblast-like synovial (FLS) cells in the joint synovium, resulting in local tissue hypoxia and inflammatory infiltration. Recent studies show that the cellular metabolism supports cell proliferation and differentiation and thus affects cell function, which is called immunometabolism (Cui et al., 2022). Immunometabolism has been actively studied in the field of tumor, and various types of metabolic modalities have been analyzed and outlined for different types of malignancies. Whereas, studies in RA have not been as extensive.

The RA microenvironment is characterized by low oxygen, acidity, and low nutrient concentrations (Gaber et al., 2005). In pathological states, almost all metabolic pathways, such as gluconeogenesis, lipid, and protein metabolism, exhibit different metabolic activation patterns (Masoumi et al., 2020). Metabolic reprogramming, with some positive correlation between the activation of the aggressive phenotype of FLS cells and subsequent destruction of bone and cartilage (de Oliveira et al., 2019), has a hallmark position in explaining pathogenic cell function and differentiation in RA (Weyand and Goronzy, 2017). As certain metabolic pathways or metabolites can be exploited to trigger abnormal activation of immune cells or stromal cells involved in RA (Okano et al., 2018), regulating immune metabolism or correcting metabolic abnormalities are other ground-breaking treatment strategies for RA.

Conventional, biological, and new non-biological disease-modifying antirheumatic drugs, are frequently used to treat patients with RA. Although the majority of patients now have a positive outlook, several patients still prove treatment resistance. New therapies are so desperately needed (Smolen et al., 2016). Therefore, identifying and creating innovative, efficient, and safer options for long-term use is necessarily needed. More than 75% of the world’s population continues to employ phytotherapy, a traditional medicinal technique, to cure a variety of ailments (Koycheva et al., 2023).

Here, we present the detailed pathogenesis of RA involving immunometabolism processes of glycolysis, lipid, amino acid and nucleotide metabolism. We further summarize natural products as prospective clues for the development of effective therapeutic approaches and how to treat RA by targeting key enzymes or regulating metabolic balance.

## 2 Imbalanced immune metabolism and corresponding therapeutic natural products in RA

### 2.1 Glucose metabolism

Carbohydrate metabolism is the main metabolism that provides biomass and energy after cell activation. During the physiological state, glucose can maintain a dynamic equilibrium of several biomass. Glucose metabolism mainly includes three types of pathways: glucose, which is first transported into the cell through glucose transporter 1 (GLUT1), then broken down by sequential metabolic enzymes, including hexokinase (HK), aldolase, phosphoglycerate kinase 1 (PGK1), and pyruvate kinase, to generate pyruvate, undergoes glycolysis in the cytoplasm to produce lactic acid under oxygen-deficient conditions; pyruvate is transported into the mitochondria under oxygen-enriched conditions and converted into acetyl-CoA in the tricarboxylic acid cycle (TCA cycle). The generated NADH and FADH are further oxidatively phosphorylated (OXPHOS) to generate ATP; and pentose phosphate pathway results in biomass generation. Resting-state cellular metabolism is dominated by OXPHOS.

#### 2.1.1 Lactic acid generation pathway

##### 2.1.1.1 Lactic acid in RA pathology

**FIGURE 1:**
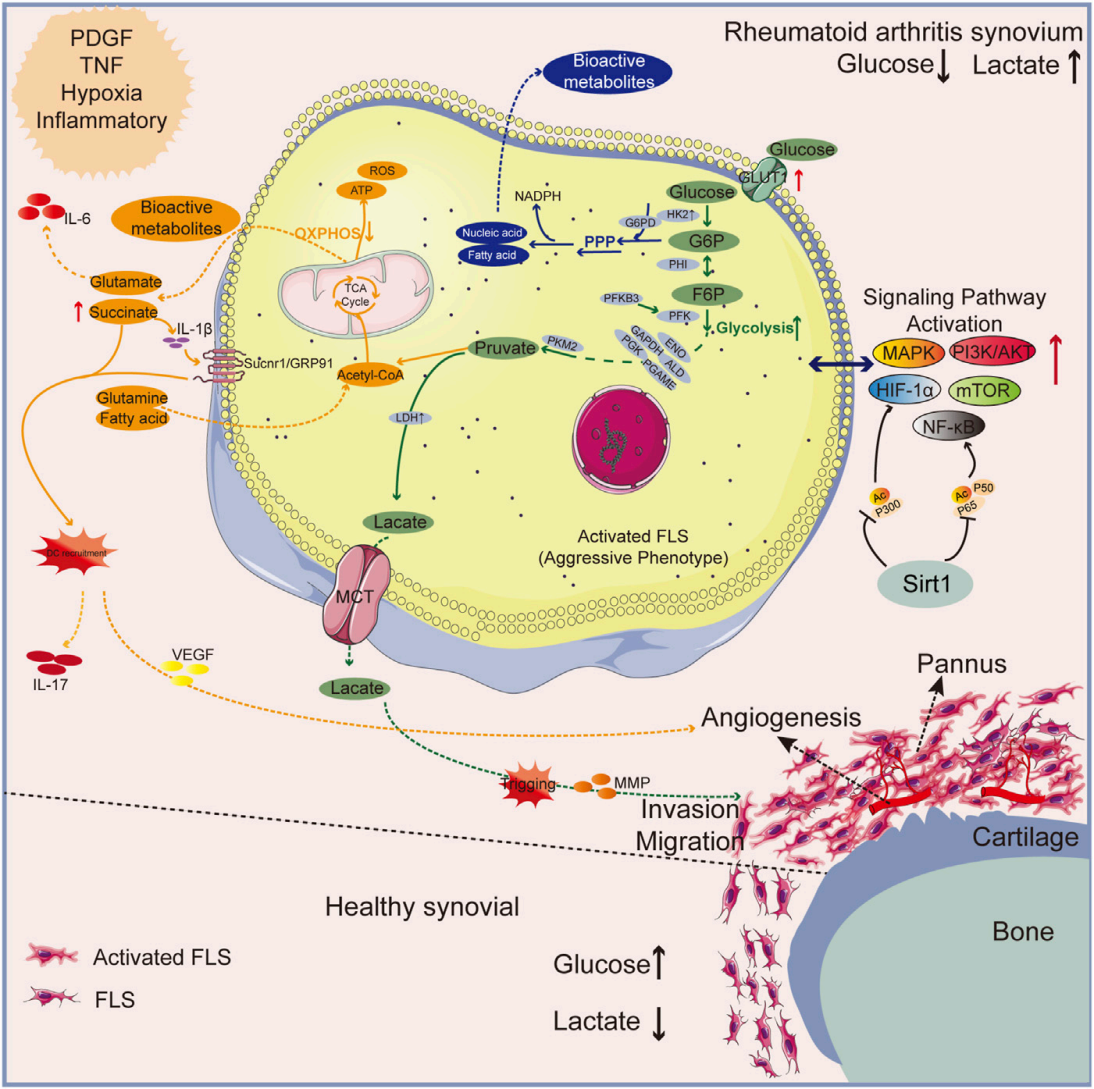
An overview of the mechanisms behind glycolysis of RA pathology. The normal synovial lining has only 2-3 layers of FLS cells; RA’s synovium is a proliferative lining rich in multiple layers of activated FLS cells and phagocytes. Upregulated GLUT1 and HK2 enzymes in synovial fluid owing to the inflammatory microenvironment results in enhanced glycolysis and pentose phosphate pathway (PPP pathway) shunt to produce bioactive metabolites. Lactic acid and succinic acid, by-products of glucose metabolism, can cause angiogenesis and bone destruction. Green arrow: glycolytic pathway; Blue arrow: PPP pathway. Orange arrow: OXPHOS pathway.

In RA synovial tissue, platelet-derived growth factors (PDGF), tumor necrosis factor (TNF), and pro-inflammatory mediators can transform resting FLS cells into an aggressive phenotype associated with glycolysis (RAFLS), known as the “Warburg effect” (Biniecka et al., 2016; Garcia-Carbonell et al., 2016; Masoumi et al., 2020). Hypoxia and high expression of HIF-1α in RA can stimulate the metabolic shift of RAFLS cells from oxidative phosphorylation to glycolysis as HIF-1α can upregulate genes involved in glycolysis, such as GLUT-1, HK-2, and LDH (Masoumi et al., 2020). ENO1 is a rate-limiting enzyme in glycolysis. Under hypoxic conditions, ENO1 gene expression was significantly upregulated in RAFLS and promoted its proliferation. The PGK1 catalyzes the transformation of 1,3-diphosphoglycerate into 3-phosphoglycerate, which results in the production of the first adenosine triphosphate in glycolysis (Zhao et al., 2016). Thus, GLUT1 and HK2 are important modulators of the glucose metabolism. Other glycolysis-related rate-limiting enzymes include, but are not limited to, PFKB3 and PKM2, which have garnered remarkable attention as potential anti-arthritis treatments (Bustamante et al., 2018). Silent information regulator 1 (SIRT1) is an energy sensor that controls associated signals like peroxisome proliferator-activated receptor-γ (PPAR-γ) and maintains metabolic homeostasis; along with deacetylates some signals to prevent their transcriptional activity, like NF-κB and HIF-1α, thereby affecting RA (Wu et al., 2021). Patients with RA exhibit SIRT1 deficiency (Wendling et al., 2014), and the SIRT1 is a negative regulator of glycolysis and M1 macrophage polarization (Lei et al., 2021). Enhancement of the glycolytic metabolic pathway leads to an increase in the lactic acid level. Moreover, further transport of lactic acid into joint lumen can stimulate the phenotype transformation, proliferation, and aggressive metastasis of FLS cells, and increases glycolysis as to accelerate the development of RA, ultimately trigging matrix metalloproteinases and further promoting bone and cartilage destruction (Bottini and Firestein, 2013; Garcia-Carbonell et al., 2016; Masuko, 2022; Pucino et al., 2023) (See green arrow route in Figure 1).

##### 2.1.1.2 Natural products targeting lactic acid production

As a natural naphthoquinone isolated from *Lithospermum erythrorhizon*, shikonin alleviated local joint swelling, and lowered lactate levels in peripheral blood of rats in an AA animal model experiment, along with less toxicity to the liver and kidneys. Mechanistically, shikonin could regulate glycolysis and ATP production by inhibiting GLUT1, PKM2 and HK2 alone, which was verified by knocking down PKM2 expression in RAFLSs. Shikonin has the ability to deactivate mTOR, phosphorylated PI3K and AKT to inhibit PI3K/AKT/mTOR pathway and the primary byproduct of glycolysis, lactate (Lu et al., 2018; Li et al., 2021). Shikonin possesses anti-inflammatory and immunomodulatory properties, but this is the first time proposed that its treatment of RA is related by regulating glycolysis. And there seems to be no relevant literature exploring the efficacy of its combination therapy.

Epigallocatechin-3-gallate (EGCG) from green tea has been demonstrated to have therapeutic efficacy in the treatment of IL-1 receptor antagonist knockdown (IL-1RaKO) autoimmune arthritis rats when administered at 40 mg/kg. In fact, mechanistic studies have found that EGCG administration inhibits the production of indicators of glycolysis and mTOR signaling pathway that support Th17 development, including HIF-1a, GLUT-1, MCT4, LDH-a, and GPI, which contributed to the amelioration of arthritis (Yang et al., 2014). Methotrexate is limited by potential hepatotoxicity in the treatment of RA, and EGCG has been identified to improve MTX-induced hepatotoxicity (Pradhan et al., 2023).

An aberrant metabolism environment of increase in PKM enzyme and lactate expression, a considerable decline in triglycerides and pyruvate were reshaped by resveratrol, a SIRT1 agonist, thereby reducing improper glycolytic metabolism to alleviate symptoms of AIA rats at a dose of 50 mg/kg (Wang DD. et al., 2022). Mechanism studies suggest that the therapeutic effect of SIRT1 may be related to the change of monocyte polarization. Overexpression of SIRT1 dramatically boosts p-AMPK expression while suppressing the aberrant increase in PKM1/2 expression. Simultaneously, it significantly inhibits the expression of HIF-1α and its downstream TPI1, nevertheless, new research has demonstrated that resveratrol induced SIRT1 activation inhibits glycolytic facilitated angiogenesis in RA, irrespective of HIF-1α (Jiang TT. et al., 2023). According to certain research, SIRT1 is typically under-expressed in RA patients. Still, some research has cast doubt on this. Therefore, the development of SIRT1-targeted anti-rheumatic therapy depends on the successful confirmation of this issue. In other research reports, resveratrol has long been considered a potential antioxidant for the treatment of RA, which improves RA by activating the SIRT1-Nrf2 signaling pathway (Wang et al., 2020). Although resveratrol has excellent clinical efficacy, patients with multiple myeloma have reported major side effects (Berman et al., 2017).

α-mangostin extracted from mangosteen can effectively inhibit aerobic glycolysis, restore the level of LDH, and abrogate HIF-1α/VEGF expression in AIA rats when the dose is set at 30 mg/kg/day, thereby alleviating inflammation-related hypoxia. This property of α-mangostin is beneficial in blocking synovial pathologic neovascularization (Jiang et al., 2021). There was a more notable reduction in VEGF in the synovium when α-mangostin therapy was employed in place of the typical DMARD leflunomide. This promising discovery suggested that α-mangostin could be more effective than conventional anti-rheumatic medications in regulating angiogenesis (Zuo et al., 2018; Jiang et al., 2021). In addition to these, in AIA models, α-mangostin has been provided to prevent M1 polarization of macrophages and monocytes by concurrently upregulating SIRT1 and PPAR-γ (Wu et al., 2023), and also to reduce inflammation by interfering with the metabolism-immune feedback system mediated by fat cells (Hu et al., 2021). α-mangostin has been demonstrated to be safe in a variety of animal studies. However, increased doses or repeated dosing should be considered for probable adverse effects such as dysbiosis, cardiac insufficiency, and liver toxicity (Do and Cho, 2020).

#### 2.1.2 Succinate generation pathway

##### 2.1.2.1 Succinate in RA pathology

High levels of ROS and inflammatory mediators boost glycolysis in M1 macrophages, which causes a buildup of intermediates from the TCA cycle, particularly succinate (Peyssonnaux et al., 2007; West et al., 2011; Mills et al., 2016). By producing IL-1β, succinate has a role in recruiting dendritic cells to lymphoid tissue. Th17 cell releasing factors are activated when succinate upregulates the succinate receptor, Sucnr1/GRP91, intensifying the inflammatory milieu (Littlewood-Evans et al., 2016; Saraiva et al., 2018) and inducing VEGF expression, which results in abnormal angiogenesis (Reddy et al., 2018). Furthermore, inflammation promotes the synthesis of the physiologically active metabolite itaconate, which hinders succinate dehydrogenase’s (SDH) ability to operate as an enzyme and causes succinate buildup. When SDH is blocked, the TCA cycle is disrupted, which encourages the generation of ROS inside the mitochondria, the stabilization of HIF-1α, and the prolonged fermentation of glycolysis within the activated cells (Lampropoulou et al., 2016). (See orange arrow route in Figure 1).

##### 2.1.2.2 Natural products targeting succinate production

Cinnamon is a TCM used to treat RA. Cinnamaldehyde has been found to alleviate the advancement of RA by modulating the succinate/GPR91/HIF-1α axis to limit NLRP3 activation and IL-1β release when administered at 200 mg/kg *in vivo* and 12.5 μM *in vitro* (Liu et al., 2020). Although cinnamaldehyde modifies the generation of succinic acid, its major mechanism of attenuating collagen-induced arthritis is via lowering pro-inflammatory cytokines and free radicals (Mateen et al., 2019). For this reason, cinnamaldehyde primarily serves as an adjuvant in the management of RA.

TGF-β1 stimulation of hypoxia increased succinic acid buildup in the synovium of RA rats by reversing SDH activation, and it also triggered the activation of the NLRP3 inflammasome in a way that was dependent on HIF-1α induction. Clematichinenoside AR, a compound isolated from the root of *Clematis manshurica* Rupr., inhibits TGF-β1-induced hypoxia and the activation of succinic acid-associated NLRP3 inflammasome in the synovium of RA rats by inhibiting SDH activity, thereby preventing myofibroblast activation via the prevention of a cross-talk between inflammation and fibrosis whose dose at 50 mg/kg (Li et al., 2016). Accordingly, succinate as a metabolic signal could be one of the targets for RA intervention.

#### 2.1.3 Intermediate metabolites generation pathway

Additionally, there are other pathologic phenomena in the pathway that triggers RA “Warburg effect” leading to activation of lipid and protein metabolism. Glutamate is elevated in arthritic joints, and the triggering of glutamate receptors induces the release of IL-6 and arthritic pain (Bonnet et al., 2015) (See orange arrow route in Figure 1). Fatty acid and nucleic acid are examples of intermediate metabolites also regulate signaling pathways that trigger immune cells to pick up various signals in the microenvironment and drive pathogenic actions (Weyand and Goronzy, 2017) (See blue arrow route in Figure 1). We go over the related natural products in the section below.

### 2.2 Lipid metabolism

To meet the body’s energy requirements, the synthesis and breakdown of lipids in the body maintain a homeostatic balance. Lipid metabolism is involved in the process of cartilage synthesis (Robinson et al., 2022). However, the RA synovial tissue’s inflammatory milieu induces a dysfunctional state of lipid metabolism. A prior study demonstrated a strong correlation between the presence of inflammatory mediators in the serum of RA patients and CIA mice and lipid metabolism (Arias de la Rosa et al., 2018). Preliminary studies in the 1960s showed a 40%–60% increase in phospholipids and cholesterol in synovial fluid in RA patients compared to corresponding serum samples from healthy controls (Bole, 1962). Phospholipids (categorized as glycerophospholipids and sphingolipids), cholesterol, triglycerides, and plasma lipoproteins account for the majority of biochemical components of post-synthesis fat metabolism.

#### 2.2.1 Choline pathway

##### 2.2.1.1 Choline kinase–a tried enzyme in RA

**FIGURE 2:**
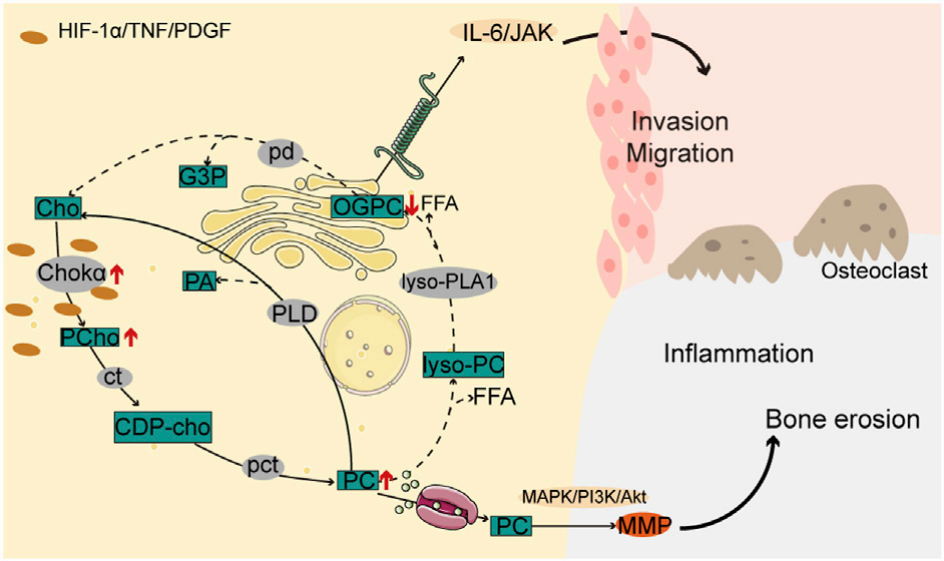
Choline pathway is involved in the pathogenesis of RA. In RAFLS cells, increased choline kinase activity promotes phosphocholine and phosphatidylcholine biosynthesis while reducing glycerophosphocholine metabolites, resulting in MAPK/PI3K/Akt and IL-6/JAK pathway activation along with increased inflammation, and malignant cell invasion and migration, and bone erosion.

With the aggravation of inflammation, the choline pathway, where choline kinase (Chokα) catalyzes the conversion of choline into CDP-choline and subsequent formation of phosphatidylcholine (PC), is highly activated in FLS, resulting in the activation of the regulatory MAPK/PI3K/Akt pathway, MMP expression, and promotion of invasive metastasis of RAFLS cells (Glunde et al., 2011; Guma et al., 2015; Bustamante et al., 2017; Miriam Jose and Rasool, 2022). Correspondingly, the metabolite, 1-oleoyl-sn-glycero-3-phosphocholine (OPGC), in the inhibited choline phospholipid metabolism pathway is downregulated in the plasma of RA patients and participates in the pathogenesis of RA by modulating the IL-6/JAK signaling pathway (Figure 2) (Su et al., 2022). The glycerophospholipid metabolic pathway is markedly dysregulated in RA patients, according to multi-omics research. Acetylcholinesterase (ACHE), one of the key genes, showed a substantial enrichment in the glycerophospholipid pathway. Glycerophosphocholine and inflammatory characteristics have a substantial negative association (Jian et al., 2023).

##### 2.2.1.2 Natural products targeting choline pathway

Network pharmacological studies have shown that *Tripterygium wilfordii* glycosides (TWG) can target PLA2 and thus interfere with glycerophospholipid metabolism and ether lipid metabolism, resulting in changes in lysoPC and PC levels. The reduction of lyso-PC levels in CIA rats can be corrected treatment with 6 mg/kg TWG per day (Gao et al., 2023). ChoKα has been reported to regulate the tumor-like phenotype of RA-FLS via activating MAPK and PI3K/Akt proteins. In the IL-21-induced tumor-like phenotype assay of AIA-FLS cells, myricetin, a flavonoid, significantly inhibited the high proliferative and invasive potential of AIA-FLSs at an administered concentration of 10 μM. Mechanistically, myricetin inhibited the IL-21/Ras/ChoKα signaling cascade response (Jose and Rasool, 2023).

#### 2.2.2 Sphingolipid metabolism

##### 2.2.2.1 Sphingolipid metabolism in RA pathology

**FIGURE 3:**
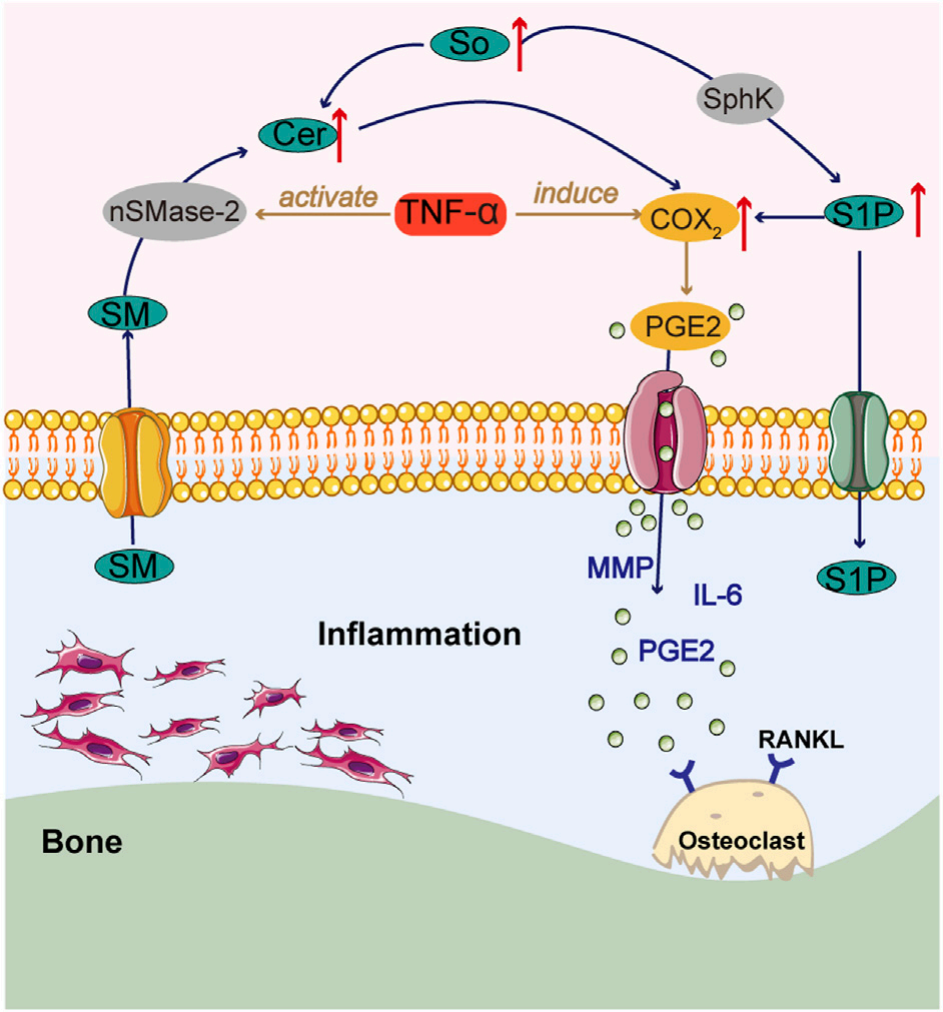
Summary chart of sphingolipid metabolism in RA pathology.

Lipids are known to contribute to the progression of chronic inflammation, and of the many lipids formed, sphingolipids are thought to be the most deleterious, with their ability to affect bone homeostasis (Seal et al., 2024). Sphingosine (So), sphingosine 1-phosphate (S1P), and ceramide (Cer) are the central molecules of sphingolipid metabolism (Meshcheryakova et al., 2017). Cer promotes chondrocyte apoptosis (Herr and Debatin, 2001). The balance between them is hypothesized to act as a switch that can control whether cells grow or die (Cuvillier et al., 1996). Sphingosine kinase (SphK) phosphorylates So to S1P (Bustamante et al., 2017), and the synovial fluid of RA patients has been found to contain active SphK and high amounts of S1P (Kitano et al., 2006). Two SphK/S1P pathway-regulated mechanisms may be simultaneously involved in the development of RA: inflammation and bone catabolism. S1P stimulates the migration and invasion of FLS, causing synovial thickening and promoting osteoclast formation and synovitis (El Jamal et al., 2020). Under inflammatory conditions, TNF-α activates neutral sphingomyelinase-2 (nSMase-2), resulting in the hydrolysis of sphingomyelin (SM) to Cer, and activates ceramidase (CDase) and SphK, resulting in increased levels of Cer and S1P. The buildup of Cer and S1P promotes the expression of COX-2 and the release of PGE2, which are involved in the pathogenesis of RA, and enhances the MMP response, leading to accelerated bone destruction (Figure 3) (Qu et al., 2018).

##### 2.2.2.2 Natural products targeting sphingolipid metabolism

According to a study, treatment with *T. wilfordii* glycosides (TWG, 6 mg/kg) entirely corrected the high Cer levels and markedly decreased the levels of several SM compounds, which has a positive therapeutic effect on RA and inflammation. CDase is required for the conversion of Cer to SM. Some data suggest that TWG can interact with CDase to modulate Cer levels (Zhu et al., 2022). Nevertheless, a growing number of research investigations have demonstrated that *T. wilfordii*’s active components frequently have adverse consequences including damage of the skin, gastrointestinal, reproductive, blood, and hepatobiliary systems (Lin et al., 2020). Geniposide extracted from *Gardenia jasminoides* Ellis, when administered 60 mg/kg daily by gavage, can reverse the levels of Cer, palmitoyl ethanolamine, and glycerophospholipid metabolites in AA rats and further reduce inflammation in AA rats by regulating imbalanced sphingolipid metabolism (Zhan et al., 2020; Ke et al., 2022). Changes in geniposide regulated Cer levels were associated with downregulation of A-SMase expression. In addition, geniposide alleviates VEGF-induced angiogenesis by down-regulating S1P/S1PR1 signaling activation (Wang Y. et al., 2022) and attenuates RAFLS aberrant proliferation by preventing HIF-1α accumulation in synovial anoxic microenvironment (Gan et al., 2022).

#### 2.2.3 Cholesterol biosynthetic

##### 2.2.3.1 Cholesterol biosynthetic in RA pathology

**FIGURE 4:**
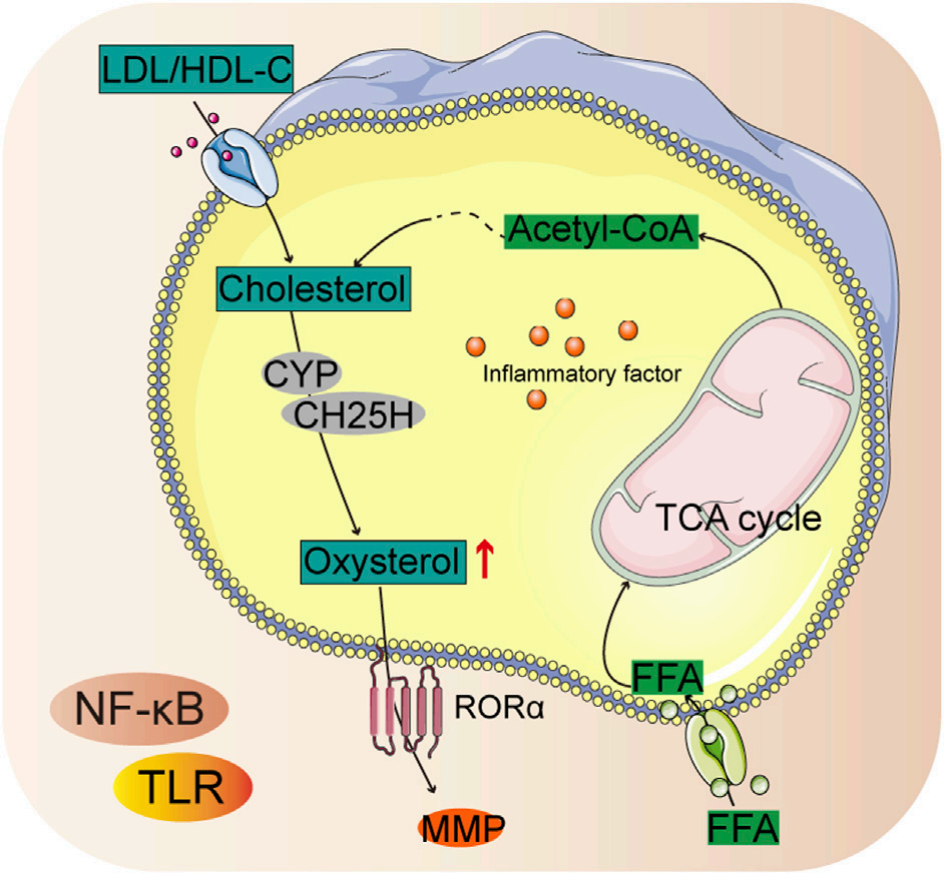
Summary chart of cholesterol biosynthetic in RA pathology.

Cholesterol biosynthetic activity is increased in chondrocytes, which promotes chondrogenesis by preventing apoptosis (Wu and De Luca, 2004). Patients with RA have upregulated cholesterol hydroxylase (CH25H) that facilitates cholesterol biosynthesis and production of more oxysterol metabolites (Perucha et al., 2019). Cellular homeostasis of cholesterol is controlled by end-product feedback mechanisms performed by the sterol regulatory element binding protein (SREBP) pathway (Yan et al., 2021). Cholesterol metabolism was found to exert catabolic functions in chondrocytes by upregulating matrix-degrading enzymes, leading to the development of arthritis (Figure 4) (Choi et al., 2019).

##### 2.2.3.2 Natural products targeting cholesterol biosynthetic

Berberine is hypothesized to suppress the inflammatory proliferation of RAFLSs by modulating AMPK/lipogenesis, LPA/LPA_1_/ERK/p38 MAPK (Shen et al., 2020), and ROS-AMPK signaling pathways at 80 μM. TNF-induced RAFLSs overexpress lipid metabolism regulator, SREBP1. Of note, berberine alleviates this expression, thereby reducing the intracellular TNF-stimulated inflammatory proliferation of RAFLSs via mitigating palmitic acid levels (Fan et al., 2018). On the other hand, additional research indicates that the antiarthritic effect of berberine is related to the inhibition of glycolysis. Berberine activates AMPK signaling, downregulates mTORC1/HIF-1α signaling and inhibits the glycolysis of M1 macrophages (Cheng et al., 2023). Berberine reversed the glycolytic reprogramming of CD4^+^ T cells induced by M1-exo (Cai et al., 2024). Although it has previously been found to cross the placenta to cause harmful effects on the fetus and lead to uterine contractions and miscarriage, berberine has not been found to show any cytotoxic or mutagenic effects at the clinical dosages utilized (Bansod et al., 2021). Recent studies have revealed that certain clinical anti-RA medications are used in conjunction with berberine to reduce side effects. To take one example, the combination of berberine and diclofenac sodium can lessen damage to the intestinal mucosa (Huang et al., 2021).

#### 2.2.4 Fatty acids metabolism

##### 2.2.4.1 Fatty acids metabolism in RA pathology

**FIGURE 5:**
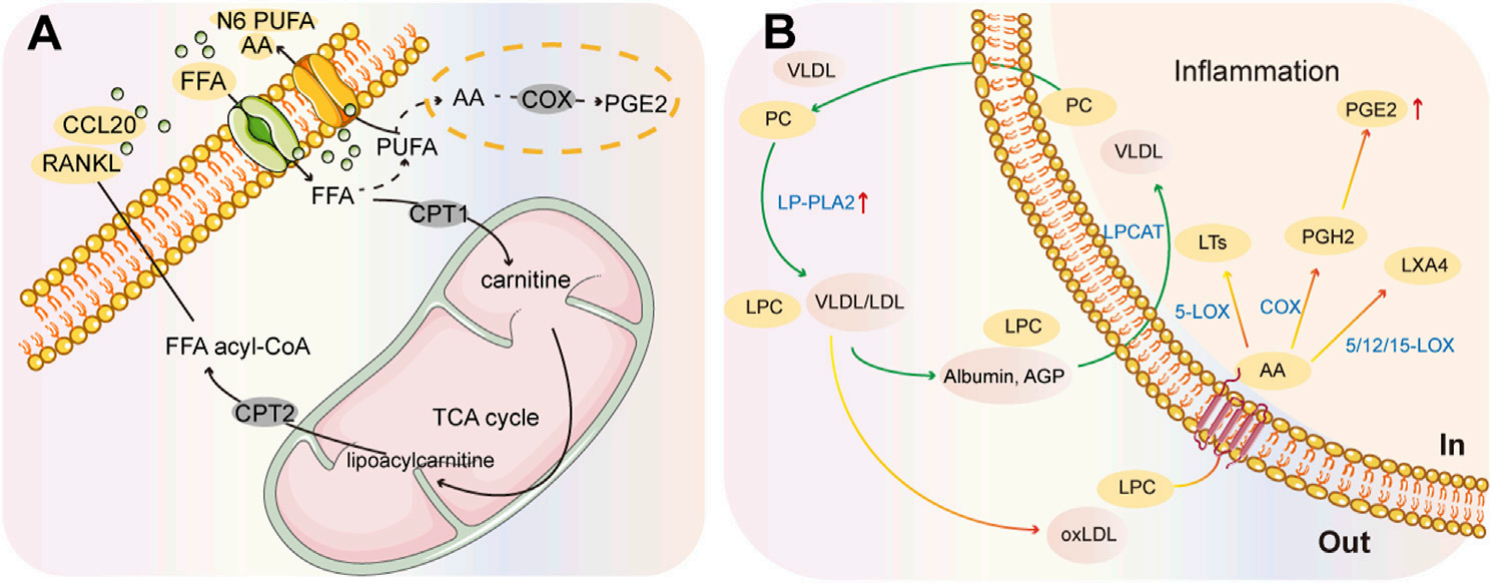
Summary chart of fatty acid metabolism in RA pathology. **(A)** CCL20-mediated inflammatory cascade response. **(B)** PC/PLA/LPC metabolic axis. Phospholipases (PLA) can generate LPC from PC and release fatty acids from membrane phospholipids. These fatty acids can be metabolized into inflammatory lipid mediators, such as prostaglandins and PGE2, by different cyclooxygenases (COX) and lipoxygenases (LOX).

Free fatty acids are present in the serum and synovial fluid of RA patients, and can impact joint tissues and immune cells involved in the pathophysiology of the disease. According to a study, the synovial environment stimulates carnitine abundance in the mitochondrial FAO pathway of human monocytes under hypoxic conditions, which can trigger a CCL20-mediated inflammatory cascade promoting RA pathogenesis (Rodgers et al., 2020). Fatty acids can be metabolized into bioactive lipid mediators, such as pro-inflammatory prostaglandins (PGs), thromboxanes, and leukotrienes (LTs), that can further damage bone and cartilage, and contribute to the pro-inflammatory environment of FLS in RA (Figure 5A) (Lei et al., 2023). Moreover, RA patients have higher activation of PLA enzymes than healthy individuals, which may lead to higher levels of fatty acids and pro-inflammatory effects of the PC/PLA/LPC axis. Studies have shown that PGE2 and the enzymes involved in its production, PLA2 and COX, are more highly expressed in synovial fluid (Figure 5B) (Brouwers et al., 2015). Arachidonic acid (AA) metabolism is involved in the synthesis of PGs and LTs through cyclooxygenase (COX) and lipoxygenase (LOX) to stimulate inflammatory responses (Figure 5B). Recent clinical results suggest that those individuals at risk for ACPA-positive RA show higher levels of LOX-derived oxylipins (O'Neil et al., 2024).

PUFA is one of the metabolite fatty acids of triglycerides that is divided into N6 PUFA with a pro-inflammatory effect and N3 PUFA with an anti-inflammatory effect based on the location and role of the double bond. N3 PUFA can regulate gene expression by activating the peroxisome PPAR-γ (Sun et al., 2008). PPAR-γ has been discovered in rat and human cartilage, and activating PPAR-γ prevents human chondrocytes from producing NO and MMP-13 in response to IL-1 (Fahmi et al., 2001; Yang et al., 2021). Thus, PPAR-γ ligands may be of therapeutic value in RA (Ricote et al., 1998).

##### 2.2.4.2 Natural products targeting inflammatory fatty acids

Thymoquinone (TQ) has been demonstrated to prevent RA by suppressing COX, LOX, inflammatory cytokines, LPO, and NO (Ali et al., 2021). Thymoquinone has regulatory effects on inflammatory molecular signaling pathways like NF-κβ, JAK-STAT, MAPK signaling, immune cells, and epigenetic machinery (Vaillancourt et al., 2011).

Caffeic acid is a potent inhibitor of LT biosynthesis that blocks 5-LOX activity and AA release, thereby improving RA through its anti-inflammatory and anti-lipid peroxidation actions (Boudreau et al., 2012). Furthermore, caffeic acid ameliorate adjuvant-induced arthritis in rats via targeting inflammatory signals, chitinase-3-like protein-1 and angiogenesis at 50 mg/kg/day for 20 days. Notably, caffeic acid has similar efficacy to celecoxib in alleviating assisted induced arthritis with fewer side effects. These findings may advocate the use of caffeic acid as an adjunct strategy for RA management (Fikry et al., 2019).

An earlier experiment revealed that norisoboldine (NOR), isolated from *Linderae Radix*, was effective at alleviating arthritic rat conditions when administered orally at a dose of 30 mg/kg. Fang et al. proposed that the anti-arthritic effects of NOR were attributed to the restoration of aberrant lipid metabolism, as demonstrated in detail by the upregulation of carnitine palmitoyltransferase 1 expression and downregulation of fatty acid synthase expression by NOR in the spleen and synovial tissues of CIA rats (Fang et al., 2021).

Isosilybin A, a major bioactive constituent of silymarin, is a partial PPARγ agonist that modifies abnormalities of lipid metabolism and stimulates cholesterol efflux from THP-1 macrophages (Wang et al., 2015). Likewise, Morin (Miao et al., 2022) and ginsenoside Rg1 (Zhang et al., 2017) is medications with PPARγ agonist characteristic that can alleviate RA.

The anti-rheumatic effects of TWG may possibly be attributed to the suppression of pro-inflammatory lipid mediators but the stimulation of certain pro-resolving mediators, and the 5-LOX is a direct target of TWG (Zhang et al., 2021). According to an analysis of the chemical constituents of TWG, 5R-Hydroxytriptolide (LLDT-8) is a novel analog of *T. wilfordii*-derived triptolide. LLDT-8 regulates the expression of stearoyl-CoA desaturase-1 and PPARγ, which contributes to the promotion of significant degradation of lipids and the inhibition of lipid synthesis. *T. wilfordii* glycosides have been demonstrated to reduce the inflammatory response by down-regulating PGs, metalloproteinases, and inducible COX-2 in previous investigations (Shan et al., 2023). Despite the fact that these results validate TWG’s advantageous characteristics in treating RA, there is still cause for worry given TWG’s widely recognized toxicity as seen in clinical studies.

#### 2.2.5 Lipid peroxidation

Fatty acid oxidative (FAO) metabolism is involved in the mechanism of joint destruction in RA (Huang et al., 2022a). Lipid peroxidation might be the defining mechanism of the damage that occurs during RA. Hesperidin (Umar et al., 2013) and quercetin exhibits anti-inflammatory and antioxidant effects in the treatment of arthritis (Jeyadevi et al., 2013). And there are no significant toxicological effects of hesperidin and quercetin were found in cell and animal toxicological evaluation experiments (Ortiz-Andrade et al., 2020). Similarly, compounds, such as protocatechuic acid (Lende et al., 2011) and the extracts of *Trigonella foenum graecum* (Fenugreek) seeds (Suresh et al., 2012) can minimize lipid peroxidation to treat arthritis.

### 2.3 Amino acid metabolism

#### 2.3.1 Amino acid metabolism in RA pathology

Glutamine is a potential biomarker in RA patients as it regulates RAFLS proliferation and is involved in the pathogenesis of RA. In RAFLSs, glutaminase 1 expression increased and glutamine metabolism increased (Takahashi et al., 2017). Ornithine is a precursor of glutamate associated with RA; thus, elevated levels of ornithine may affect TNF-α expression via glutamate activity.

Additionally, patients with RA exhibit several other amino acid metabolism, including abnormal tryptophan (Rylance, 1969) and arginine metabolism (Panfili et al., 2020). Metabolomic profiles of synovial fluid from RA patients revealed the downregulation of tryptophan metabolites, which is attributed to the aberrant methylation of the cytotoxic T lymphocyte antigen 4 gene promoter and a hypoxic environment in synovial fluid leading to indoleamine 2,3-dioxygenase 1 (IDO1) low expression (Kaul et al., 2020; Panfili et al., 2020). Tryptophan is converted to kynurenine (KYNA) by the IDO1 enzyme, and IDO1-mediated tryptophan depletion suppresses T helper cells to terminate inappropriate immunological reactions (Tykocinski et al., 2017). The researchers have now conducted tryptophan pathway metabolomics analysis on serum samples from RA patients and normal individuals. Higher serotonin pathway metabolite concentrations and lower kynurenine and indole pathway metabolite concentrations were linked to RA. Quinolinic acid concentrations were higher in RA patients than in healthy individuals within the kynurenine pathway, but kynurenic acid concentrations and the ratio of xanthurenic acid to its precursor 3-hydroxykynurenine were lower. AADAT, also known as kynurenine aminotransferase 2, catalyzes the production of kynurenic acid and xanthurenic acid from precursors of quinolinic acid. Compared to healthy persons, patients with early RA had significantly reduced serum concentrations of AADAT (Phillips, 2024). The latest study has found that RA patients have reduced serum levels of KYNA and xanthurenic, as well as indole derivatives, and increased levels of quinolinic acid. Mechanistically, quinolinic acid favors the proliferation of RAFLSs and affects their cellular metabolism by inducing mitochondrial respiration and glycolysis (Moulin et al., 2024).

Synovial fluid from RA patients has high levels of arginase 1 (Arg1), the rate-limiting enzyme of arginine metabolism, which promotes fibrosis and tissue regeneration (Munder, 2009). iNOS and Arg1 play a major role in controlling the intracellular metabolism of L-arginine (L-Arg) in macrophages. L-Arg catalyzes the conversion of iNOS into NO and L-citrulline. L-citrulline has been discovered as a biomarker for RA (Yang et al., 2020). Early onset of RA is associated with dysregulation of citrullination, a process catalyzed by peptidylarginine deiminase isoform 4 (PADI-4) (Yang et al., 2024). Studies have shown that platelet arginase activity is greater in CIA rats than in control rats, which leads to an inadequate supply of substrate (L-arginine), resulting in nitric oxide synthase isolation, thereby increasing oxidative stress and lipid peroxidation and promoting the development of arthritis (Prati et al., 2011). Based on a latest study, L-Arg helps inflammatory osteoclasts convert glycolysis to oxidative phosphorylation. This process increases ATP synthesis, purine metabolism, and inosine and hypoxanthine levels, which helps to reduce bone erosion and arthritis (Cao et al., 2024).

#### 2.3.2 Natural products targeting amino acid metabolism

A clinical trial evaluated the correlation between serum ornithine levels in RA patients and their degree of remission after the administration of sinomenine (SIN, 120 mg, twice daily), and revealed the potential therapeutic mechanism by which SIN could upregulate the gene expression level of Arg1, thereby inducing a reduction in arginine and further downregulation of ornithine (Shi et al., 2022). Another study found that SIN substantially elevated tryptophan metabolites, stimulated aryl hydrocarbon receptors (AhR) in CIA rats, and regulated Th17/Treg balance (Jiang ZM. et al., 2023). SIN can cause gastrointestinal reactions and transient allergic reactions (Huang et al., 2022b). Furthermore, a meta-analysis of ten randomized controlled trials revealed that a combined therapy of MTX and SIN was more successful than MTX alone (Chen et al., 2015). MTX and SIN combination treatment had a therapeutic effectiveness in prior research that was similar to MTX plus leflunomide, but with fewer side effects (Huang et al., 2019).

Eugenol, a phenolic substance, may inhibit pro-inflammatory arthritic responses in arthritis rats by regulating arginase in platelets and preventing lipid peroxidation when administered 2.5 mg/kg orally (Adefegha et al., 2018). At a certain concentration, eugenol impaired the locomotor ability of *Drosophila melanogaster* (Julio Cesar Silva, 2022).

A paper confirms that inhibition of PADI-4 by vitamin B12 ameliorates RA in CAIA mice (Yang et al., 2024). Atractylodes oil as a novel anti-RA drug (Linghang et al., 2020), and licorice flavonoids (Wei et al., 2018) have regulatory effects on a variety of amino acid metabolites.

### 2.4 Nucleotide metabolism and 1C metabolism

Adenosine is produced by intracellular ATP catabolism and rises in hypoxic and inflammatory environments. The purine metabolism enzyme adenosine deaminase (ADA) influences both joint inflammation and adenosine levels. Patients with RA have higher levels of ADA activity in their synovial fluid, and findings indicate a substantial positive association between MMP-9 and ADA isoforms (Atta et al., 2024). According to previous studies, quercetin has an inhibitory effect on lymphocyte ADA activity and modulates nucleoside triphosphate diphosphohydrolase (NTPDase) activity to reduce adenosine levels (Saccol et al., 2019). According to a new concept, from participation in purine biosynthesis to post-translational modification of proteins, 1C metabolism promotes cell development and differentiation through the methionine and folate cycle. The regulation of immune cell function and differentiation, including t-cells, b-cells, and macrophages, is directly affected by 1C metabolism, and thus 1C metabolism could be a determinant of RA, promising to be explored for therapeutic intervention (Dang et al., 2024).

## 3 Discussion and conclusions

Glycolysis, lipid metabolism, amino acid biosynthesis, and nucleotide metabolism catalyze the disease process to some extent, making metabolic reprogramming of practical importance in the management of RA. This review sought to focus on the products or regulators of metabolic processes that are involved in the disruption of the internal environment to stimulate synovial cytopathology and the natural products that are available to improve or treat arthritis through metabolic reprogramming pathways. Numerous significant metabolic substances and processes, including glycolysis and phenylalanine metabolism, have been the subject of recent research. Studies relating to RA and nucleotide metabolism, still, have not been thoroughly examined. Natural products, as metabolic therapies for RA, can reverse internal environmental metabolic disturbances from multiple angles and targets, with compatibility patterns that rarely cause toxic side effects. The main information was summarized, as shown in Table 1. The rate-limiting enzymes of gluconeogenesis are GLUT1, HK2, G6PD, PFKB3, PKM2, and LDH. Tan et al. (2022) provided a list of organic compounds that might interfere with glycolysis-related transporter proteins and rate-limiting enzymes, which are primarily evaluated for malignancies. In a sense, these natural products may be potential agents that inhibit metabolic reprogramming in the development of RA.

**TABLE 1:** Effect of natural products on different immune metabolism in rheumatoid arthritis.

Categories		Natural product	Experiment models	Mechanisms	Ref.
glucose metabolism	lactic acid generation	shikonin	RA-FLSs	PKM2, GLUT1, HK2, PI3K/AKT/mTOR, lactic acid↓	Li et al. (2021); Lu et al. (2018)
			AA rats		
		epigallocatechin-3-gallate	IL-1RaKO mice	HIF-1a, GLUT-1, MCT4, LDH-α, GPI↓	Yang et al. (2014)
		resveratrol	AIA rats	AMPK/SIRT1 signaling↑	Wang et al. (2022a)
		α-mangostin	AIA rats	LDH, SIRT1, PPAR-γ↑	Jiang et al. (2021)
				HIF-1α/VEGF↓	Wu et al. (2023)
	succinate generation	cinnamaldehyde	AA rats	succinate/GRP91/HIF-1α, NLRP3, IL-1β↓	Liu et al. (2020)
		clematichinenoside AR	CIA rats	SDH, NLRP3↓	Li et al. (2016)
lipid metabolism	choline pathway	*Tripterygium wilfordii* glycosides	CIA rats	lysoPC, PC↓	Gao et al. (2023)
		myricetin	AIA-FLSs	IL-21/Ras/ChoKα signaling↓	Jose and Rasool (2023)
	sphingolipid metabolism	*Tripterygium wilfordii* glycosides	CIA rats	Cer, SM↓	Zhu et al. (2022)
		geniposide	AA rats	Cer, A-SMase, S1P/S1PR1, HIF-1α↓	Wang et al. (2022b)
					Gan et al. (2022)
	cholesterol biosynthetic	berberine	RAFLSs	SREBP1, LPA/LPA1/ERK/p38 MAPK, mTORC1/HIF-1α↓	Fan et al. (2018)
					Cheng et al. (2023)
	fatty acids metabolism	thymoquinone	inflammatory mouse	COX、LOX↓	Vaillancourt et al. (2011)
				NF-κβ、JAK-STAT、MAPK	
		caffeic acid	AA rats	5-LOX, AA↓	Boudreau et al. (2012)
		norisoboldine	CIA rats	CPT-1↑, FASN↓	Fang et al. (2021)
		isosilybin A	THP-1 macrophages	PPARγ↑	Wang et al. (2015)
		morin	CIA rats	PPARγ↑	Miao et al. (2022)
		ginsenoside Rg1	AIA rats	PPARγ↑	Zhang et al. (2017)
		*Tripterygium wilfordii* glycosides	CIA rats	5-LOX, COX-2↓, PPARγ↑	Zhang et al. (2021)
					Shan et al. (2023)
	lipid peroxidation	Hesperidin	CIA rats	free radical load, neutrophil activation and infiltration↓	Umar et al. (2013)
		quercetin	AIA rats	ABTS, DPPH, NO, ROS↓	Jeyadevi et al. (2013)
amino acid metabolism	ornithine	sinomenine	RA patients	Arg1↑	Shi et al. (2022)
	tryptophan			Th17/Treg balance↑	Jiang et al. (2023b)
	arginine				
		eugenol	carrageenan induced rats	Arg1↑	Adefegha et al. (2018)
nucleotide metabolism	adenosine	quercetin	lymphocyte	MMP-9, ADA↓	Saccol et al. (2019)

The treatment of RA with Western medicine still entails severe side effects and drug resistance. In the treatment of RA, alternative medicine is becoming increasingly unique. We believe that the role of natural products in immune metabolism modulation in the therapy of RA can give clinicians with novel advice.

## 4 Perspectives and challenges

Cellular metabolism is an integral biochemical process in the pathogenesis of RA. However, few new mechanistic and clinical studies have been conducted on different metabolic treatment targets, which remain in the early stages of evaluation. Despite the fact that there are several descriptions of metabolic problems in RA, most of them focus on a single metabolism and are insufficiently detailed and clustered. The metabolic regulation processes of herbs in RA have not been completely studied, and the majority of them have only been assessed by metabolomics. Target metabolomics should be employed more in a subsequent study to confirm and investigate these compounds. The majority of the research on metabolomics investigations of bioactive natural products in the treatment mechanisms of RA does not address or clarify whether they have hazardous side effects, which is crucial and necessitates a more comprehensive evaluation including additional clinical trials. Notably, most conventional medications lack specificity, and new therapeutic approaches and research emphasize alternative medicine pathways that can be more precisely targeted to reduce inflammation while preventing the negative metabolic consequences of conventional therapy. Deficiencies of natural drugs, such as short half-life in the human body and susceptibility to cumulative toxicity, can be significantly improved by structural modification using computer-aided drug design and encapsulation of nanomaterials.

## Figures and Tables

**Table:** 

**RA**	Rheumatoid arthritis
**FLS cells**	fibroblast-like synovial cells
**GLUT1**	glucose transporter 1
**HK**	hexokinase
**PGK1**	phosphoglycerate kinase
**TCA cycle**	tricarboxylic acid cycle
**OXPHOS**	oxidative phosphorylation
**LDH**	lactate dehydrogenase
**PDGF**	platelet-derived growth factors
**TNF**	tumor Necrosis Factor
**PPAR-γ**	peroxisome proliferator-activated receptor-γ
**VEGF**	vascular endothelial growth factor
**SDH**	succinate dehydrogenase
**PPP**	pentose phosphate pathway
**G6P**	glucose-6-phosphate
**F6P**	fructose-6-phosphate
**LPLAT**	Lysophospholipid acyltransferase
**OPGC**	1-oleoyl-sn-glycero-3-phosphocholine
**Chokα**	choline kinase
**PC**	phosphatidylcholine
**ACHE**	Acetylcholinesterase
**So**	Sphingosine
**S1P**	sphingosine 1-phosphate
**Cer**	ceramide
**SphK**	Sphingosine kinase
**nSMase-2**	sphingomyelinase-2
**CDase**	ceramidase
**CH25H**	cholesterol hydroxylase
**PGs**	prostaglandins
**LTs**	leukotrienes
**AA**	arachidonic acid
**COX**	cyclooxygenase
**LOX**	lipoxygenase
**FAO**	fatty acid oxidation
**IDO1**	indoleamine 2,3-dioxygenase 1
**KYNA**	kynurenine
**AADAT**	kynurenine aminotransferase 2
**Arg1**	arginase 1
**L-Arg**	L-arginine
**PADI-4**	peptidylarginine deiminase isoform 4
**SIN**	sinomenine
**Sirt1**	Silent information regulator 1
**NOR**	norisoboldine

## References

[B1] Adefegha S. A. Okeke B. M. Oboh G. Ijomone O. M. Oyeleye S. I. (2018). Modulatory effect of eugenol on arginase, nucleotidase, and adenosine deaminase activities of platelets in a carrageenan-induced arthritis rat model: a possible anti-arthritic mechanism of eugenol. Biomed. Pharmacother. 106, 1616–1623. 10.1016/j.biopha.2018.07.143 30119237

[B2] Ali M. Y. Akter Z. Mei Z. Zheng M. Tania M. Khan M. A. (2021). Thymoquinone in autoimmune diseases: therapeutic potential and molecular mechanisms. Biomed. Pharmacother. 134, 111157. 10.1016/j.biopha.2020.111157 33370631

[B3] Arias De La Rosa I. Escudero-Contreras A. Rodríguez-Cuenca S. Ruiz-Ponce M. Jiménez-Gómez Y. Ruiz-Limón P. (2018). Defective glucose and lipid metabolism in rheumatoid arthritis is determined by chronic inflammation in metabolic tissues. J. Intern Med. 284, 61–77. 10.1111/joim.12743 29532531

[B4] Atta A. Salem M. M. El-Said K. S. Mohamed T. M. (2024). Mechanistic role of quercetin as inhibitor for adenosine deaminase enzyme in rheumatoid arthritis: systematic review. Cell. Mol. Biol. Lett. 29, 14. 10.1186/s11658-024-00531-7 38225555 PMC10790468

[B5] Bansod S. Saifi M. A. Godugu C. (2021). Molecular updates on berberine in liver diseases: bench to bedside. Phytother. Res. 35, 5459–5476. 10.1002/ptr.7181 34056769

[B6] Berman A. Y. Motechin R. A. Wiesenfeld M. Y. Holz M. K. (2017). The therapeutic potential of resveratrol: a review of clinical trials. NPJ Precis. Oncol. 1, 35. 10.1038/s41698-017-0038-6 28989978 PMC5630227

[B7] Biniecka M. Canavan M. Mcgarry T. Gao W. Mccormick J. Cregan S. (2016). Dysregulated bioenergetics: a key regulator of joint inflammation. Ann. Rheum. Dis. 75, 2192–2200. 10.1136/annrheumdis-2015-208476 27013493 PMC5136702

[B8] Bole G. G. (1962). Synovial fluid lipids in normal individuals and patients with rheumatoid arthritis. Arthritis Rheum. 5, 589–601. 10.1002/art.1780050606 13968590

[B9] Bonnet C. S. Williams A. S. Gilbert S. J. Harvey A. K. Evans B. A. Mason D. J. (2015). AMPA/kainate glutamate receptors contribute to inflammation, degeneration and pain related behaviour in inflammatory stages of arthritis. Ann. Rheum. Dis. 74, 242–251. 10.1136/annrheumdis-2013-203670 24130267 PMC4283694

[B10] Bottini N. Firestein G. S. (2013). Duality of fibroblast-like synoviocytes in RA: passive responders and imprinted aggressors. Nat. Rev. Rheumatol. 9, 24–33. 10.1038/nrrheum.2012.190 23147896 PMC3970924

[B11] Boudreau L. H. Maillet J. Leblanc L. M. Jean-François J. Touaibia M. Flamand N. (2012). Caffeic acid phenethyl ester and its amide analogue are potent inhibitors of leukotriene biosynthesis in human polymorphonuclear leukocytes. PLoS One 7, e31833. 10.1371/journal.pone.0031833 22347509 PMC3276500

[B12] Brouwers H. Von Hegedus J. Toes R. Kloppenburg M. Ioan-Facsinay A. (2015). Lipid mediators of inflammation in rheumatoid arthritis and osteoarthritis. Best. Pract. Res. Clin. Rheumatol. 29, 741–755. 10.1016/j.berh.2016.02.003 27107510

[B13] Bustamante M. F. Garcia-Carbonell R. Whisenant K. D. Guma M. (2017). Fibroblast-like synoviocyte metabolism in the pathogenesis of rheumatoid arthritis. Arthritis Res. Ther. 19, 110. 10.1186/s13075-017-1303-3 28569176 PMC5452638

[B14] Bustamante M. F. Oliveira P. G. Garcia-Carbonell R. Croft A. P. Smith J. M. Serrano R. L. (2018). Hexokinase 2 as a novel selective metabolic target for rheumatoid arthritis. Ann. Rheum. Dis. 77, 1636–1643. 10.1136/annrheumdis-2018-213103 30061164 PMC6328432

[B15] Cai W. W. Gao Y. Cheng J. W. Yu Y. Zong S. Y. Li Y. H. (2024). Berberine modulates the immunometabolism and differentiation of CD4(+) T cells alleviating experimental arthritis by suppression of M1-exo-miR155. Phytomedicine 124, 155255. 10.1016/j.phymed.2023.155255 38181528

[B16] Cao S. Li Y. Song R. Meng X. Fuchs M. Liang C. (2024). L-arginine metabolism inhibits arthritis and inflammatory bone loss. Ann. Rheum. Dis. 83, 72–87. 10.1136/ard-2022-223626 37775153 PMC10803985

[B17] Chen X. M. Huang R. Y. Huang Q. C. Chu Y. L. Yan J. Y. (2015). Systemic review and meta-analysis of the clinical efficacy and adverse effects of zhengqing fengtongning combined with methotrexate in rheumatoid arthritis. Evid. Based Complement. Altern. Med. 2015, 910376. 10.1155/2015/910376 PMC4561327 26379753

[B18] Cheng J. W. Yu Y. Zong S. Y. Cai W. W. Wang Y. Song Y. N. (2023). Berberine ameliorates collagen-induced arthritis in mice by restoring macrophage polarization via AMPK/mTORC1 pathway switching glycolytic reprogramming. Int. Immunopharmacol. 124, 111024. 10.1016/j.intimp.2023.111024 37827054

[B19] Choi W. S. Lee G. Song W. H. Koh J. T. Yang J. Kwak J. S. (2019). The CH25H-CYP7B1-RORα axis of cholesterol metabolism regulates osteoarthritis. Nature 566, 254–258. 10.1038/s41586-019-0920-1 30728500

[B20] Cui H. R. Zhang J. Y. Cheng X. H. Zheng J. X. Zhang Q. Zheng R. (2022). Immunometabolism at the service of traditional Chinese medicine. Pharmacol. Res. 176, 106081. 10.1016/j.phrs.2022.106081 35033650

[B21] Cuvillier O. Pirianov G. Kleuser B. Vanek P. G. Coso O. A. Gutkind S. (1996). Suppression of ceramide-mediated programmed cell death by sphingosine-1-phosphate. Nature 381, 800–803. 10.1038/381800a0 8657285

[B22] Dang S. Jain A. Dhanda G. Bhattacharya N. Bhattacharya A. Senapati S. (2024). One carbon metabolism and its implication in health and immune functions. Cell. Biochem. Funct. 42, e3926. 10.1002/cbf.3926 38269500

[B23] De Oliveira P. G. Farinon M. Sanchez-Lopez E. Miyamoto S. Guma M. (2019). Fibroblast-like synoviocytes glucose metabolism as a therapeutic target in rheumatoid arthritis. Front. Immunol. 10, 1743. 10.3389/fimmu.2019.01743 31428089 PMC6688519

[B24] Do H. T. T. Cho J. (2020). Mangosteen pericarp and its bioactive xanthones: potential therapeutic value in alzheimer's disease, Parkinson's disease, and depression with pharmacokinetic and safety profiles. Int. J. Mol. Sci. 2110, 6211. 10.3390/ijms21176211 PMC7504283 32867357

[B25] El Jamal A. Bougault C. Mebarek S. Magne D. Cuvillier O. Brizuela L. (2020). The role of sphingosine 1-phosphate metabolism in bone and joint pathologies and ectopic calcification. Bone 130, 115087. 10.1016/j.bone.2019.115087 31648078

[B26] Fahmi H. Di Battista J. A. Pelletier J. P. Mineau F. Ranger P. Martel-Pelletier J. (2001). Peroxisome proliferator--activated receptor gamma activators inhibit interleukin-1beta-induced nitric oxide and matrix metalloproteinase 13 production in human chondrocytes. Arthritis Rheum. 44, 595–607. 10.1002/1529-0131(200103)44:3<595::aid-anr108>3.0.co;2-8 11263774

[B27] Fan X. X. Leung E. L. Xie Y. Liu Z. Q. Zheng Y. F. Yao X. J. (2018). Suppression of lipogenesis via reactive oxygen species-AMPK signaling for treating malignant and proliferative diseases. Antioxid. Redox Signal 28, 339–357. 10.1089/ars.2017.7090 28665143

[B28] Fang Y. Duan C. Zhang J. Dai Y. Xia Y. (2021). NMR-based untargeted metabolomics approach to investigate the systemic lipid metabolism regulation of norisoboldine in collagen-induced arthritis rats. Eur. J. Pharmacol. 912, 174608. 10.1016/j.ejphar.2021.174608 34743982

[B29] Fikry E. M. Gad A. M. Eid A. H. Arab H. H. (2019). Caffeic acid and ellagic acid ameliorate adjuvant-induced arthritis in rats via targeting inflammatory signals, chitinase-3-like protein-1 and angiogenesis. Biomed. Pharmacother. 110, 878–886. 10.1016/j.biopha.2018.12.041 30562713

[B30] Gaber T. Dziurla R. Tripmacher R. Burmester G. R. Buttgereit F. (2005). Hypoxia inducible factor (HIF) in rheumatology: low O2! See what HIF can do. Ann. Rheum. Dis. 64, 971–980. 10.1136/ard.2004.031641 15800008 PMC1755583

[B31] Gan P. Sun M. Wu H. Ke J. Dong X. Chen F. (2022). A novel mechanism for inhibiting proliferation of rheumatoid arthritis fibroblast-like synoviocytes: geniposide suppresses HIF-1α accumulation in the hypoxic microenvironment of synovium. Inflamm. Res. 71, 1375–1388. 10.1007/s00011-022-01636-5 36109396

[B32] Gao Y. Qian Q. Xun G. Zhang J. Sun S. Liu X. (2023). Integrated metabolomics and network analysis reveal changes in lipid metabolisms of tripterygium glycosides tablets in rats with collagen-induced arthritis. Comput. Struct. Biotechnol. J. 21, 1828–1842. 10.1016/j.csbj.2023.02.050 36923473 PMC10009339

[B33] Garcia-Carbonell R. Divakaruni A. S. Lodi A. Vicente-Suarez I. Saha A. Cheroutre H. (2016). Critical role of glucose metabolism in rheumatoid arthritis fibroblast-like synoviocytes. Arthritis Rheumatol. 68, 1614–1626. 10.1002/art.39608 26815411 PMC4963240

[B34] Glunde K. Bhujwalla Z. M. Ronen S. M. (2011). Choline metabolism in malignant transformation. Nat. Rev. Cancer 11, 835–848. 10.1038/nrc3162 22089420 PMC4337883

[B35] Guma M. Sanchez-Lopez E. Lodi A. Garcia-Carbonell R. Tiziani S. Karin M. (2015). Choline kinase inhibition in rheumatoid arthritis. Ann. Rheum. Dis. 74, 1399–1407. 10.1136/annrheumdis-2014-205696 25274633 PMC4382461

[B36] Herr I. Debatin K. M. (2001). Cellular stress response and apoptosis in cancer therapy. Blood 98, 2603–2614. 10.1182/blood.v98.9.2603 11675328

[B37] Hu Y. H. Han J. Wang L. Shi C. Li Y. Olatunji O. J. (2021). α-Mangostin alleviated inflammation in rats with adjuvant-induced arthritis by disrupting adipocytes-mediated metabolism-immune feedback. Front. Pharmacol. 12, 692806. 10.3389/fphar.2021.692806 34305602 PMC8293671

[B38] Huang D. N. Wu F. F. Zhang A. H. Sun H. Wang X. J. (2021). Efficacy of berberine in treatment of rheumatoid arthritis: from multiple targets to therapeutic potential. Pharmacol. Res. 169, 105667. 10.1016/j.phrs.2021.105667 33989762

[B39] Huang R. Y. Pan H. D. Wu J. Q. Zhou H. Li Z. G. Qiu P. (2019). Comparison of combination therapy with methotrexate and sinomenine or leflunomide for active rheumatoid arthritis: a randomized controlled clinical trial. Phytomedicine 57, 403–410. 10.1016/j.phymed.2018.12.030 30851515

[B40] Huang Z. Luo R. Yang L. Chen H. Zhang X. Han J. (2022a). CPT1A-Mediated fatty acid oxidation promotes precursor osteoclast fusion in rheumatoid arthritis. Front. Immunol. 13, 838664. 10.3389/fimmu.2022.838664 35273614 PMC8902079

[B41] Huang Z. Mao X. Chen J. He J. Shi S. Gui M. (2022b). The efficacy and safety of zhengqing fengtongning for knee osteoarthritis: a systematic review and meta-analysis of randomized clinical trials. Evid. Based Complement. Altern. Med. 2022, 2768444. 10.1155/2022/2768444 PMC8794657 35096105

[B42] Jeyadevi R. Sivasudha T. Rameshkumar A. Ananth D. A. Aseervatham G. S. Kumaresan K. (2013). Enhancement of anti arthritic effect of quercetin using thioglycolic acid-capped cadmium telluride quantum dots as nanocarrier in adjuvant induced arthritic Wistar rats. Colloids Surf. B Biointerfaces 112, 255–263. 10.1016/j.colsurfb.2013.07.065 23994749

[B43] Jian C. Wei L. Wu T. Li S. Wang T. Chen J. (2023). Comprehensive multi-omics analysis reveals the core role of glycerophospholipid metabolism in rheumatoid arthritis development. Arthritis Res. Ther. 25, 246. 10.1186/s13075-023-03208-2 38102690 PMC10722724

[B44] Jiang T. T. Ji C. F. Cheng X. P. Gu S. F. Wang R. Li Y. (2021). α-Mangostin alleviated HIF-1α-Mediated angiogenesis in rats with adjuvant-induced arthritis by suppressing aerobic glycolysis. Front. Pharmacol. 12, 785586. 10.3389/fphar.2021.785586 34987400 PMC8721667

[B45] Jiang T. T. Ji C. L. Yu L. J. Song M. K. Li Y. Liao Q. (2023a). Resveratrol-induced SIRT1 activation inhibits glycolysis-fueled angiogenesis under rheumatoid arthritis conditions independent of HIF-1α. Inflamm. Res. 72, 1021–1035. 10.1007/s00011-023-01728-w 37016140

[B46] Jiang Z. M. Zeng S. L. Huang T. Q. Lin Y. Wang F. F. Gao X. J. (2023b). Sinomenine ameliorates rheumatoid arthritis by modulating tryptophan metabolism and activating aryl hydrocarbon receptor via gut microbiota regulation. Sci. Bull. (Beijing) 68, 1540–1555. 10.1016/j.scib.2023.06.027 37422372

[B47] Jose A. M. Rasool M. (2023). Myricetin ameliorates the IL-21-induced tumorigenic phenotype of adjuvant-induced arthritis FLS by modulating the choline kinase signaling cascade. Vitro Cell. Dev. Biol. Anim. 59, 811–820. 10.1007/s11626-023-00827-6 38032403

[B48] Julio Cesar Silva R. L. S. P. Freitas T. S.De Rocha J. E. Macedo N. S. Nonato C.De F. A. Linhares M. L. (2022). Evaluation of antibacterial and toxicological activities of essential oil of Ocimum gratissimum L. and its major constituent eugenol. Food Biosci. 50, 102128. 10.1016/j.fbio.2022.102128

[B49] Kaul N. C. Mohapatra S. R. Adam I. Tucher C. Tretter T. Opitz C. A. (2020). Hypoxia decreases the T helper cell-suppressive capacity of synovial fibroblasts by downregulating Ido1-mediated tryptophan metabolism. Rheumatol. Oxf. 59, 1148–1158. 10.1093/rheumatology/kez587 31846032

[B50] Ke J. T. Zhang H. Bu Y. H. Gan P. R. Chen F. Y. Dong X. T. (2022). Metabonomic analysis of abnormal sphingolipid metabolism in rheumatoid arthritis synovial fibroblasts in hypoxia microenvironment and intervention of geniposide. Front. Pharmacol. 13, 969408. 10.3389/fphar.2022.969408 35935818 PMC9353937

[B51] Kitano M. Hla T. Sekiguchi M. Kawahito Y. Yoshimura R. Miyazawa K. (2006). Sphingosine 1-phosphate/sphingosine 1-phosphate receptor 1 signaling in rheumatoid synovium: regulation of synovial proliferation and inflammatory gene expression. Arthritis Rheum. 54, 742–753. 10.1002/art.21668 16508938

[B52] Koycheva I. K. Marchev A. S. Stoykova I. D. Georgiev M. I. (2023). Natural alternatives targeting psoriasis pathology and key signaling pathways: a focus on phytochemicals. Phytochem. Rev. 10.1007/s11101-023-09886-9

[B53] Lampropoulou V. Sergushichev A. Bambouskova M. Nair S. Vincent E. E. Loginicheva E. (2016). Itaconate links inhibition of succinate dehydrogenase with macrophage metabolic remodeling and regulation of inflammation. Cell. Metab. 24, 158–166. 10.1016/j.cmet.2016.06.004 27374498 PMC5108454

[B54] Lei M. Tao M. Q. Wu Y. J. Xu L. Yang Z. Li Y. (2021). Metabolic enzyme triosephosphate isomerase 1 and nicotinamide phosphoribosyltransferase, two independent inflammatory indicators in rheumatoid arthritis: evidences from collagen-induced arthritis and clinical samples. Front. Immunol. 12, 795626. 10.3389/fimmu.2021.795626 35111160 PMC8801790

[B55] Lei Q. Yang J. Li L. Zhao N. Lu C. Lu A. (2023). Lipid metabolism and rheumatoid arthritis. Front. Immunol. 14, 1190607. 10.3389/fimmu.2023.1190607 37325667 PMC10264672

[B56] Lende A. B. Kshirsagar A. D. Deshpande A. D. Muley M. M. Patil R. R. Bafna P. A. (2011). Anti-inflammatory and analgesic activity of protocatechuic acid in rats and mice. Inflammopharmacology 19, 255–263. 10.1007/s10787-011-0086-4 21748471

[B57] Li J. Pang J. Liu Z. Ge X. Zhen Y. Jiang C. C. (2021). Shikonin induces programmed death of fibroblast synovial cells in rheumatoid arthritis by inhibiting energy pathways. Sci. Rep. 11, 18263. 10.1038/s41598-021-97713-6 34521930 PMC8440543

[B58] Li Y. Zheng J. Y. Liu J. Q. Yang J. Liu Y. Wang C. (2016). Succinate/NLRP3 inflammasome induces synovial fibroblast activation: therapeutical effects of clematichinenoside AR on arthritis. Front. Immunol. 7, 532. 10.3389/fimmu.2016.00532 28003810 PMC5141240

[B59] Lin N. Zhang Y. Q. Jiang Q. Liu W. Liu J. Huang Q. C. (2020). Clinical practice guideline for tripterygium glycosides/tripterygium wilfordii tablets in the treatment of rheumatoid arthritis. Front. Pharmacol. 11, 608703. 10.3389/fphar.2020.608703 33519474 PMC7845140

[B60] Linghang Q. Yiyi X. Guosheng C. Kang X. Jiyuan T. Xiong L. (2020). Effects of atractylodes oil on inflammatory response and serum metabolites in adjuvant arthritis rats. Biomed. Pharmacother. 127, 110130. 10.1016/j.biopha.2020.110130 32289576

[B61] Littlewood-Evans A. Sarret S. Apfel V. Loesle P. Dawson J. Zhang J. (2016). GPR91 senses extracellular succinate released from inflammatory macrophages and exacerbates rheumatoid arthritis. J. Exp. Med. 213, 1655–1662. 10.1084/jem.20160061 27481132 PMC4995082

[B62] Liu P. Wang J. Wen W. Pan T. Chen H. Fu Y. (2020). Cinnamaldehyde suppresses NLRP3 derived IL-1β via activating succinate/HIF-1 in rheumatoid arthritis rats. Int. Immunopharmacol. 84, 106570. 10.1016/j.intimp.2020.106570 32413739

[B63] Lu B. Wang Z. Ding Y. Wang X. Lu S. Wang C. (2018). RIP1 and RIP3 contribute to shikonin-induced glycolysis suppression in glioma cells via increase of intracellular hydrogen peroxide. Cancer Lett. 425, 31–42. 10.1016/j.canlet.2018.03.046 29608987

[B64] Masoumi M. Mehrabzadeh M. Mahmoudzehi S. Mousavi M. J. Jamalzehi S. Sahebkar A. (2020). Role of glucose metabolism in aggressive phenotype of fibroblast-like synoviocytes: latest evidence and therapeutic approaches in rheumatoid arthritis. Int. Immunopharmacol. 89, 107064. 10.1016/j.intimp.2020.107064 33039953

[B65] Masuko K. (2022). Glucose as a potential key to fuel inflammation in rheumatoid arthritis. Nutrients 14, 2349. 10.3390/nu14112349 35684149 PMC9182926

[B66] Mateen S. Shahzad S. Ahmad S. Naeem S. S. Khalid S. Akhtar K. (2019). Cinnamaldehyde and eugenol attenuates collagen induced arthritis via reduction of free radicals and pro-inflammatory cytokines. Phytomedicine 53, 70–78. 10.1016/j.phymed.2018.09.004 30668414

[B67] Meshcheryakova A. Mechtcheriakova D. Pietschmann P. (2017). Sphingosine 1-phosphate signaling in bone remodeling: multifaceted roles and therapeutic potential. Expert Opin. Ther. Targets 21, 725–737. 10.1080/14728222.2017.1332180 28524744 PMC5470107

[B68] Miao Y. Wu X. Xue X. Ma X. Yang L. Zeng X. (2022). Morin, the PPARγ agonist, inhibits Th17 differentiation by limiting fatty acid synthesis in collagen-induced arthritis. Cell. Biol. Toxicol. 39, 1433–1452. 10.1007/s10565-022-09769-3 36121554

[B69] Mills E. L. Kelly B. Logan A. Costa A. S. H. Varma M. Bryant C. E. (2016). Succinate dehydrogenase supports metabolic repurposing of mitochondria to drive inflammatory macrophages. Cell. 167, 457–470. 10.1016/j.cell.2016.08.064 27667687 PMC5863951

[B70] Miriam Jose A. Rasool M. (2022). Choline kinase: an underappreciated rheumatoid arthritis therapeutic target. Life Sci. 309, 121031. 10.1016/j.lfs.2022.121031 36206833

[B71] Moulin D. Millard M. Taïeb M. Michaudel C. Aucouturier A. Lefèvre A. (2024). Counteracting tryptophan metabolism alterations as a new therapeutic strategy for rheumatoid arthritis. Ann. Rheum. Dis. 83, 312–323. 10.1136/ard-2023-224014 38049981 PMC10894831

[B72] Munder M. (2009). Arginase: an emerging key player in the mammalian immune system. Br. J. Pharmacol. 158, 638–651. 10.1111/j.1476-5381.2009.00291.x 19764983 PMC2765586

[B73] Okano T. Saegusa J. Takahashi S. Ueda Y. Morinobu A. (2018). Immunometabolism in rheumatoid arthritis. Immunol. Med. 41, 89–97. 10.1080/25785826.2018.1531186 30938274

[B74] O'neil L. J. Anaparti V. Winter T. Smolik I. Meng X. Aukema H. M. (2024). Lipoxygenase-derived oxylipins are enriched in anti-citrullinated protein antibody (ACPA)-positive individuals at risk for developing rheumatoid arthritis. Arthritis Res. Ther. 26, 51. 10.1186/s13075-024-03274-0 38360827 PMC10868017

[B75] Ortiz-Andrade R. Araujo-León J. A. Sánchez-Recillas A. Navarrete-Vazquez G. González-Sánchez A. A. Hidalgo-Figueroa S. (2020). Toxicological screening of four bioactive citroflavonoids: *in vitro*, *in vivo*, and *in silico* approaches. Molecules 25, 5959. 10.3390/molecules25245959 33339310 PMC7766697

[B76] Panfili E. Gerli R. Grohmann U. Pallotta M. T. (2020). Amino acid metabolism in rheumatoid arthritis: friend or foe?. Biomolecules 10, 1280. 10.3390/biom10091280 32899743 PMC7563518

[B77] Perucha E. Melchiotti R. Bibby J. A. Wu W. Frederiksen K. S. Roberts C. A. (2019). The cholesterol biosynthesis pathway regulates IL-10 expression in human Th1 cells. Nat. Commun. 10, 498. 10.1038/s41467-019-08332-9 30700717 PMC6353904

[B78] Peyssonnaux C. Cejudo-Martin P. Doedens A. Zinkernagel A. S. Johnson R. S. Nizet V. (2007). Cutting edge: essential role of hypoxia inducible factor-1alpha in development of lipopolysaccharide-induced sepsis. J. Immunol. 178, 7516–7519. 10.4049/jimmunol.178.12.7516 17548584

[B79] Phillips R. (2024). Manipulating tryptophan metabolism in arthritis. Nat. Rev. Rheumatol. 20, 67. 10.1038/s41584-024-01077-w 38216756

[B80] Pradhan A. Sengupta S. Sengupta R. Chatterjee M. (2023). Attenuation of methotrexate induced hepatotoxicity by epigallocatechin 3-gallate. Drug Chem. Toxicol. 46, 717–725. 10.1080/01480545.2022.2085738 35698845

[B81] Prati C. Berthelot A. Wendling D. Demougeot C. (2011). Endothelial dysfunction in rat adjuvant-induced arthritis: up-regulation of the vascular arginase pathway. Arthritis Rheum. 63, 2309–2317. 10.1002/art.30391 21484767

[B82] Pucino V. Nefla M. Gauthier V. Alsaleh G. Clayton S. A. Marshall J. (2023). Differential effect of lactate on synovial fibroblast and macrophage effector functions. Front. Immunol. 14, 1183825. 10.3389/fimmu.2023.1183825 37304267 PMC10251493

[B83] Qu F. Zhang H. Zhang M. Hu P. (2018). Sphingolipidomic profiling of rat serum by UPLC-Q-TOF-MS: application to rheumatoid arthritis study. Molecules 2310, 1324. 10.3390/molecules23061324 PMC6099492 29857511

[B84] Reddy P. H. Manczak M. Yin X. Grady M. C. Mitchell A. Tonk S. (2018). Protective effects of Indian spice curcumin against amyloid-β in alzheimer's disease. J. Alzheimers Dis. 61, 843–866. 10.3233/jad-170512 29332042 PMC5796761

[B85] Ricote M. Li A. C. Willson T. M. Kelly C. J. Glass C. K. (1998). The peroxisome proliferator-activated receptor-gamma is a negative regulator of macrophage activation. Nature 391, 79–82. 10.1038/34178 9422508

[B86] Robinson G. Pineda-Torra I. Ciurtin C. Jury E. C. (2022). Lipid metabolism in autoimmune rheumatic disease: implications for modern and conventional therapies. J. Clin. Invest 13210, e148552. 10.1172/JCI148552 PMC8759788 35040437

[B87] Rodgers L. C. Cole J. Rattigan K. M. Barrett M. P. Kurian N. Mcinnes I. B. (2020). The rheumatoid synovial environment alters fatty acid metabolism in human monocytes and enhances CCL20 secretion. Rheumatol. Oxf. 59, 869–878. 10.1093/rheumatology/kez378 31497857

[B88] Rylance H. J. (1969). Hypertaurinuria in rheumatoid arthritis. Ann. Rheum. Dis. 28, 41–44. 10.1136/ard.28.1.41 5786281 PMC1010494

[B89] Saccol R. Da Silveira K. L. Adefegha S. A. Manzoni A. G. Da Silveira L. L. Coelho A. P. V. (2019). Effect of quercetin on E-NTPDase/E-ADA activities and cytokine secretion of complete Freund adjuvant-induced arthritic rats. Cell. Biochem. Funct. 37, 474–485. 10.1002/cbf.3413 31365139

[B90] Saraiva A. L. Veras F. P. Peres R. S. Talbot J. De Lima K. A. Luiz J. P. (2018). Succinate receptor deficiency attenuates arthritis by reducing dendritic cell traffic and expansion of T(h)17 cells in the lymph nodes. Faseb J. 32, 6550–6558. 10.1096/fj.201800285 29894669

[B91] Seal A. Hughes M. Wei F. Pugazhendhi A. S. Ngo C. Ruiz J. (2024). Sphingolipid-induced bone regulation and its emerging role in dysfunction due to disease and infection. Int. J. Mol. Sci. 2510, 3024. 10.3390/ijms25053024 PMC10932382 38474268

[B92] Shan Y. Zhao J. Wei K. Jiang P. Xu L. Chang C. (2023). A comprehensive review of Tripterygium wilfordii hook. f. in the treatment of rheumatic and autoimmune diseases: bioactive compounds, mechanisms of action, and future directions. Front. Pharmacol. 14, 1282610. 10.3389/fphar.2023.1282610 38027004 PMC10646552

[B93] Shen P. Jiao Y. Miao L. Chen J. H. Momtazi‐Borojeni A. A. (2020). Immunomodulatory effects of berberine on the inflamed joint reveal new therapeutic targets for rheumatoid arthritis management. J. Cell. Mol. Med. 24, 12234–12245. 10.1111/jcmm.15803 32969153 PMC7687014

[B94] Shi Y. Pan H.-D. Wu J.-L. Zou Q.-H. Xie X.-Y. Li H.-G. (2022). The correlation between decreased ornithine level and alleviation of rheumatoid arthritis patients assessed by a randomized, placebo-controlled, double-blind clinical trial of sinomenine. Engineering 16, 93–99. 10.1016/j.eng.2021.04.014

[B95] Smolen J. S. Aletaha D. Mcinnes I. B. (2016). Rheumatoid arthritis. Lancet 388, 2023–2038. 10.1016/s0140-6736(16)30173-8 27156434

[B96] Su J. Li S. Chen J. Jian C. Hu J. Du H. (2022). Glycerophospholipid metabolism is involved in rheumatoid arthritis pathogenesis by regulating the IL-6/JAK signaling pathway. Biochem. Biophys. Res. Commun. 600, 130–135. 10.1016/j.bbrc.2022.02.003 35219101

[B97] Sun H. Berquin I. M. Owens R. T. O'flaherty J. T. Edwards I. J. (2008). Peroxisome proliferator-activated receptor gamma-mediated up-regulation of syndecan-1 by n-3 fatty acids promotes apoptosis of human breast cancer cells. Cancer Res. 68, 2912–2919. 10.1158/0008-5472.can-07-2305 18413760 PMC3686510

[B98] Suresh P. Kavitha Ch N. Babu S. M. Reddy V. P. Latha A. K. (2012). Effect of ethanol extract of Trigonella foenum graecum (Fenugreek) seeds on Freund's adjuvant-induced arthritis in albino rats. Inflammation 35, 1314–1321. 10.1007/s10753-012-9444-7 22395729

[B99] Takahashi S. Saegusa J. Sendo S. Okano T. Akashi K. Irino Y. (2017). Glutaminase 1 plays a key role in the cell growth of fibroblast-like synoviocytes in rheumatoid arthritis. Arthritis Res. Ther. 19, 76. 10.1186/s13075-017-1283-3 28399896 PMC5387190

[B100] Tan C. Li L. Han J. Xu K. Liu X. (2022). A new strategy for osteoarthritis therapy: inhibition of glycolysis. Front. Pharmacol. 13, 1057229. 10.3389/fphar.2022.1057229 36438808 PMC9685317

[B101] Tykocinski L. O. Lauffer A. M. Bohnen A. Kaul N. C. Krienke S. Tretter T. (2017). Synovial fibroblasts selectively suppress Th1 cell responses through Ido1-mediated tryptophan catabolism. J. Immunol. 198, 3109–3117. 10.4049/jimmunol.1600600 28264972

[B102] Umar S. Kumar A. Sajad M. Zargan J. Ansari M. M. Ahmad S. (2013). Hesperidin inhibits collagen-induced arthritis possibly through suppression of free radical load and reduction in neutrophil activation and infiltration. Rheumatol. Int. 33, 657–663. 10.1007/s00296-012-2430-4 22527139

[B103] Vaillancourt F. Silva P. Shi Q. Fahmi H. Fernandes J. C. Benderdour M. (2011). Elucidation of molecular mechanisms underlying the protective effects of thymoquinone against rheumatoid arthritis. J. Cell. Biochem. 112, 107–117. 10.1002/jcb.22884 20872780

[B104] Wang D. D. He C. Y. Wu Y. J. Xu L. Shi C. Olatunji O. J. (2022a). AMPK/SIRT1 deficiency drives adjuvant-induced arthritis in rats by promoting glycolysis-mediated monocytes inflammatory polarization. J. Inflamm. Res. 15, 4663–4675. 10.2147/jir.s378090 35996683 PMC9392262

[B105] Wang G. Xie X. Yuan L. Qiu J. Duan W. Xu B. (2020). Resveratrol ameliorates rheumatoid arthritis via activation of SIRT1-Nrf2 signaling pathway. Biofactors 46, 441–453. 10.1002/biof.1599 31883358

[B106] Wang L. Rotter S. Ladurner A. Heiss E. H. Oberlies N. H. Dirsch V. M. (2015). Silymarin constituents enhance ABCA1 expression in THP-1 macrophages. Molecules 21, E55. 10.3390/molecules21010055 26729088 PMC4748397

[B107] Wang Y. Wu H. Gui B. J. Liu J. Rong G. X. Deng R. (2022b). Geniposide alleviates VEGF-induced angiogenesis by inhibiting VEGFR2/PKC/ERK1/2-mediated SphK1 translocation. Phytomedicine 100, 154068. 10.1016/j.phymed.2022.154068 35358930

[B108] Wei M. Ma Y. Liu Y. Zhou Y. Men L. Yue K. (2018). Urinary metabolomics study on the anti-inflammation effects of flavonoids obtained from Glycyrrhiza. J. Chromatogr. B Anal. Technol. Biomed. Life Sci. 1086, 1–10. 10.1016/j.jchromb.2018.04.007 29654981

[B109] Wendling D. Vidon C. Khan K. A. Guillot X. Godfrin-Valnet M. Abbas W. (2014). AB0116 sirt-1 activity in PBMC from patients with rheumatoid arthritis. Ann. Rheumatic Dis. 73, 842. 10.1136/annrheumdis-2014-eular.1584

[B110] West A. P. Brodsky I. E. Rahner C. Woo D. K. Erdjument-Bromage H. Tempst P. (2011). TLR signalling augments macrophage bactericidal activity through mitochondrial ROS. Nature 472, 476–480. 10.1038/nature09973 21525932 PMC3460538

[B111] Weyand C. M. Goronzy J. J. (2017). Immunometabolism in early and late stages of rheumatoid arthritis. Nat. Rev. Rheumatol. 13, 291–301. 10.1038/nrrheum.2017.49 28360422 PMC6820517

[B112] Wu S. De Luca F. (2004). Role of cholesterol in the regulation of growth plate chondrogenesis and longitudinal bone growth. J. Biol. Chem. 279, 4642–4647. 10.1074/jbc.M305518200 14612457

[B113] Wu Y. J. Fang W. J. Pan S. Zhang S. S. Li D. F. Wang Z. F. (2021). Regulation of Sirt1 on energy metabolism and immune response in rheumatoid arthritis. Int. Immunopharmacol. 101, 108175. 10.1016/j.intimp.2021.108175 34689102

[B114] Wu Y. J. Zhang S. S. Yin Q. Lei M. Wang Q. H. Chen W. G. (2023). α-Mangostin inhibited M1 polarization of macrophages/monocytes in antigen-induced arthritis mice by up-regulating silent information regulator 1 and peroxisome proliferators-activated receptor γ simultaneously. Drug Des. Devel Ther. 17, 563–577. 10.2147/dddt.S397914 PMC9969869 36860800

[B115] Yan R. Cao P. Song W. Li Y. Wang T. Qian H. (2021). Structural basis for sterol sensing by Scap and Insig. Cell. Rep. 35, 109299. 10.1016/j.celrep.2021.109299 34192549

[B116] Yang C. W. Hsu H. Y. Lee Y. Z. Lee S. J. (2024). Vitamin B12 inhibits peptidylarginine deiminases and ameliorates rheumatoid arthritis in CAIA mice. Biochem. Biophys. Res. Commun. 704, 149668. 10.1016/j.bbrc.2024.149668 38401303

[B117] Yang E. J. Lee J. Lee S. Y. Kim E. K. Moon Y. M. Jung Y. O. (2014). EGCG attenuates autoimmune arthritis by inhibition of STAT3 and HIF-1α with Th17/Treg control. PLoS One 9, e86062. 10.1371/journal.pone.0086062 24558360 PMC3928092

[B118] Yang X. Chang Y. Wei W. (2020). Emerging role of targeting macrophages in rheumatoid arthritis: focus on polarization, metabolism and apoptosis. Cell. Prolif. 53, e12854. 10.1111/cpr.12854 32530555 PMC7377929

[B119] Yang Y. Wei J. Li J. Cui Y. Zhou X. Xie J. (2021). Lipid metabolism in cartilage and its diseases: a concise review of the research progress. Acta Biochim. Biophys. Sin. (Shanghai) 53, 517–527. 10.1093/abbs/gmab021 33638344

[B120] Zhan X. Wu H. Wu H. (2020). Joint synovial fluid metabolomics method to decipher the metabolic mechanisms of adjuvant arthritis and geniposide intervention. J. Proteome Res. 19, 3769–3778. 10.1021/acs.jproteome.0c00300 32786678

[B121] Zhang K. Pace S. Jordan P. M. Peltner L. K. Weber A. Fischer D. (2021). Beneficial modulation of lipid mediator biosynthesis in innate immune cells by antirheumatic tripterygium wilfordii glycosides. Biomolecules 11, 746. 10.3390/biom11050746 34067705 PMC8155965

[B122] Zhang L. Zhu M. Li M. Du Y. Duan S. Huang Y. (2017). Ginsenoside Rg1 attenuates adjuvant-induced arthritis in rats via modulation of PPAR-γ/NF-κB signal pathway. Oncotarget 8, 55384–55393. 10.18632/oncotarget.19526 28903427 PMC5589666

[B123] Zhao Y. Yan X. Li X. Zheng Y. Li S. Chang X. (2016). PGK1, a glucose metabolism enzyme, may play an important role in rheumatoid arthritis. Inflamm. Res. 65, 815–825. 10.1007/s00011-016-0965-7 27342824

[B124] Zhu Y. Zhang L. Zhang X. Wu D. Chen L. Hu C. (2022). Tripterygium wilfordii glycosides ameliorates collagen-induced arthritis and aberrant lipid metabolism in rats. Front. Pharmacol. 13, 938849. 10.3389/fphar.2022.938849 36105231 PMC9465305

[B125] Zuo J. Yin Q. Wang Y. W. Li Y. Lu L. M. Xiao Z. G. (2018). Inhibition of NF-κB pathway in fibroblast-like synoviocytes by α-mangostin implicated in protective effects on joints in rats suffering from adjuvant-induced arthritis. Int. Immunopharmacol. 56, 78–89. 10.1016/j.intimp.2018.01.016 29367090

